# Relationship between paternal psychological distress and involvement in childcare among fathers of preschool-aged children: mediating effect of maternal psychological distress

**DOI:** 10.1186/s12887-019-1688-z

**Published:** 2019-09-03

**Authors:** Hyeon Sik Chu, Hanyi Lee

**Affiliations:** School of Nursing, Hanyang University, 222 Wangsimni-ro, Seongdong-gu, Seoul, 04763 South Korea

**Keywords:** Psychological distress, Fathers’ involvement in childcare, Preschoolers

## Abstract

**Background:**

The role of the father as a co-caregiver is becoming increasingly important across cultures. Parental psychological distress is an influencing factor of maladaptive parenting behaviors and negative psychosocial outcomes in children. Considerable research has focused on psychological distress in parents, commonly experienced during the childrearing years; however, the relationship between paternal psychological distress and fathers’ involvement in childcare has been less studied. This study aimed to examine this relationship.

**Methods:**

This study explored the relationship between parental psychological distress and fathers’ involvement in childcare by analyzing data from 1541 children and their parents from the 2011 Panel Study on Korean Children. Psychological distress was assessed using the Kessler 6-Item Psychological Distress Scale. Fathers’ involvement in childcare was measured in terms of the quality and quantity of involvement, using a Father’s Childcare Involvement Scale completed by mothers and the *daily* hours spent by fathers in *childcare*.

**Results:**

The mean scores for paternal and maternal psychological distress were 5.26 ± 4.20 and 5.79 ± 4.42, respectively; for the quality of fathers’ involvement in childcare, 14.46 ± 2.63; and for the quantity of fathers’ involvement, 2.53 ± 1.62. Paternal psychological distress was significantly correlated with maternal psychological distress and fathers’ involvement in childcare. Maternal psychological distress demonstrated a partial mediating effect on the relationship between paternal psychological distress and the quality of fathers’ involvement in childcare for preschool-aged children (β = −.085, *p* < .001); this effect was significant (Sobel test; Z = 3.13, *p* = .002). Further, maternal psychological distress demonstrated a complete mediating effect on the relationship between paternal psychological distress and the quantity of fathers’ involvement in childcare (β = −.065, *p* = .018); this effect too was significant (Sobel test; Z = 2.38, *p* = .018).

**Conclusions:**

Paternal psychological distress influenced the quality and quantity of fathers’ involvement in childcare and was mediated by maternal psychological distress. To promote fathers’ involvement in childcare, a family-centered approach for childcare should reflect the triadic interaction of father–mother–child. These findings have implications for primary health professionals, as well as policymakers who design community health programs for early childhood.

## Background

Increased social involvement by women in recent years, as well as changes in family structures, such as increased nuclearization and double-income families, have not only elevated expectations and demands for fathers’ roles as co-caregivers but also emphasized fathers’ roles in childrearing [1, 2]. As paternal involvement in childcare increases, fathers may experience tension and conflict amid the growing demands and social pressure for their roles both at work and at home.

Psychological distress refers to the emotional and psychological difficulties that affect an individual’s mental health and functioning; it is not specific to any particular DSM-5 (Diagnostic and Statistical Manual of Mental Disorders) or ICD-10 (International Statistical Classification of Diseases and Related Health Problems) diagnosis [3]. Previous studies have consistently indicated that approximately 8–10% of fathers experience psychological distress, such as depression and anxiety, in the postnatal period, which increases over time [4, 5]. A cohort study examining the incidence of parental depression in community settings among 86,957 families reported that 39% of mothers and 21% of fathers had experienced an episode of depression by the time their child was aged 12 years [6]. Psychological distress among fathers was lower than that among mothers; nevertheless, it is important to address it because it can adversely influence child development due to reduced parental interaction with the child and negative childrearing behaviors [7].

The preschool period of development is characterized by more dynamic interaction with parents as children undergo rapid language and cognitive development, and have increased initiative [8]. Fathers’ involvement in childcare during this period, therefore, contributes to the developing child’s emotional regulation ability and coping skills, and can promote emotional expression and empathy [9]. Fathers’ involvement in childcare is also important in that children may develop their gender role and ego based on their relationship with their father [10].

During a child’s development, fathers are typically more playful, while mothers are more focused on nurturing [2]. Because the father and mother have distinct roles, children can receive different types of stimulation from their parents [11, 12]. In the sense that such a difference in stimulation maximizes the effects on a child’s development, fathers’ roles are as important as mothers’ in parenting, and fathers’ unique parenting behaviors can contribute, in particular, to a child’s attachment security [1, 2, 13].

Most previous studies, however, have simplified the relationship between a father’s involvement in childcare and the child’s health outcomes, or have primarily discussed the positive impact of fathers’ involvement in childcare [2]. Similarly, regarding father–mother–child relationships, most studies have focused on understanding mothers’ psychosocial characteristics and parenting behaviors as opposed to those of fathers. In addition, the few existing studies on fathers’ psychological health are limited to investigations during the postpartum period only [14]. The present study thus aimed to examine the relationship between paternal psychological distress and fathers’ involvement in childcare as well as to identify whether maternal psychological distress has a mediating or moderating effect on this relationship.

### Study design

This study was a descriptive survey study that, through secondary data, aimed to investigate the relationship between parental psychological distress and fathers’ involvement in childcare in Korea, using the 2011 Panel Study on Korean Children (PSKC).

## Methods

### Data and participants

This study used the raw 2011 PSKC data provided by the Korea Institute of Child Care and Education (http://panel.kicce.re.kr/eng/index.jsp). Beginning in 2008, the PSKC is an annual survey of families of children born between April and July. To ensure representativeness, samples were collected via stratified sampling from 30 medical institutions in six regions nationwide, and data were collected via face-to-face interviews by trained investigators and through mail-in surveys. A total of 1754 households participated in the 2011 survey, and 1541 households with first-time married fathers with no disabilities who are living with their children were included in the final analysis. This study was approved by the Institutional Review Board at Hanyang University (HYI-17-231-2), and written informed consent was obtained from all study participants by the Korea Institute of Child Care and Education at the time of the study.

### Measures

#### Psychological distress

Parental psychological distress was measured using the Kessler 6-item Psychological Distress Scale [3], which is rated on a 5-point Likert scale, with the total score ranging from 0 to 24. This scale examines psychological distress in the past 4 weeks. A higher score indicates a higher level of psychological distress. The symptomatic cutoff point was defined as a score of 8 or more [4], indicating significant symptoms. The Cronbach’s alpha coefficient was .92 in this study.

#### Fathers’ involvement in childcare

Fathers’ involvement in childcare was assessed both qualitatively and quantitatively. The quality of fathers’ involvement in childcare was examined using a Father’s Childcare Involvement Scale completed by mothers, which was originally developed by Hong and partially modified and adapted by the PSKC staff [15]. The scale consists of four items: “My husband buys toys or other products needed by our child,” “My husband takes interest in our child’s habits and behaviors,” “My husband bathes or feeds our child food or milk,” and “My husband plays with our child frequently and talks to him/her.” Each item is scored on a 5-point Likert scale with a total score ranging from 4 to 20, with higher total scores indicating more involvement of the child’s father in childcare. The Cronbach’s alpha coefficient for these items was .73 in the study sample.

The quantity of fathers’ involvement in childcare was defined as the mean daily time (hours) that the father spends on childcare, including feeding meals or snacks, bathing, changing diapers, interacting and playing, and taking the child shopping for groceries. This metric did not include household chores, such as preparing for meals, doing laundry, and cleaning the house, or time while the child is at daycare or preschool.

#### Covariates

This study surveyed the following covariates: father’s age, education level, religion, smoking, alcohol consumption, daily working hours, parenting stress; child’s age, sex, temperament; and family household income. Father’s education level was classified into high school or lower, college, and advanced degree or higher, and religion was classified into yes and no. Alcohol consumption was classified into fewer than once per week and more than once a week, at a threshold of seven shots or five cans of beer on a single occasion, and smoking was classified into yes and no, according to current smoking status. For daily working hours, the mean hours spent on the corresponding activity by weekday was used. Fathers’ parenting stress was measured using Kim and Kang’s parenting stress scale, which consists of 11 items rated on a 5-point Likert scale, with the total score ranging from 11 to 55 [16]. A higher total score indicates a higher level of parenting stress. The Cronbach’s alpha coefficient for these items was .85 in the study sample. Family household income was divided into quantiles, and the child’s temperament was measured using the Emotionality, Activity, Sociability, and Shyness Temperament scale for children (EAS scale) [17]. In this study, only children’s emotionality (tendency to become easily upset) and activity (tendency to be restless) were measured. Five items each for the two temperaments and a 5-point rating scale was used (from 1: not characteristic or typical of your child, to 5: very characteristic or typical of your child). The scores from the items belonging to each temperament were summed to form the two temperament indicators. Higher total scores indicate a greater presence of each temperament. In this study, the Cronbach’s alpha coefficients for emotionality and activity were .72 and .78, respectively.

### Data analysis

The collected data were analyzed using the IBM SPSS Statistics 24.0 software in consideration of the complex sampling design, and weighting was applied for population estimates [18]. All participant variables were expressed by descriptive or frequency analysis. Differences in fathers’ involvement in childcare by covariates were analyzed with t-test and ANOVA. Pearson’s correlation coefficients were calculated to examine the relationship among paternal psychological distress, maternal psychological distress, and fathers’ involvement in childcare. The moderating effect of maternal psychological distress on paternal psychological distress and fathers’ involvement in childcare was analyzed using multiple linear regression analysis. Moreover, the mediating effect of maternal psychological distress on paternal psychological distress and fathers’ involvement in childcare was analyzed using Baron and Kenny’s steps for mediation [19]. A Sobel test was performed to examine whether maternal psychological distress significantly carries the influence of paternal psychological distress to fathers’ involvement in childcare [20]. Data were presented with relative frequency and were estimated by weighting (%). Statistical significance was set at α = .05.

## Results

A total of 1541 participants were included in the analysis. Table 1 shows the descriptive analysis results of demographics and study variables. The mean age of fathers was 36.24 ± 3.96 years, and 54.4% had graduated from college or had achieved an advanced degree. About 60.1% had no religion. Regarding alcohol consumption, 63.8% drank fewer than once a week, and 50.3% were current smokers. The mean daily working hours was 8.04 ± 2.27, and the mean score of fathers’ parenting stress was 27.41 ± 6.63. Family household income was divided into quantiles, and 27.4% were in the third quantile. Regarding children’s sex, 50.6% were boys, with a mean age of 38.18 ± 1.51 months. Concerning children’s temperament, the mean score for emotionality was 14.35 ± 3.06, and that for activity was 19.14 ± 3.00.

**Table 1:** Descriptive analysis of demographics and study variables (*N* = 1541)

Variable	Categories	*n*	%	M ± SD	Actual range
Father					
Age (years)				36.24 ± 3.96	22–52
Education level	High school or less	412	27.5		
	College	309	18.1		
	University or higher	820	54.4		
Religion	No	931	60.1		
	Yes	610	39.9		
Alcohol consumption	More than once a week	496	36.2		
	Less than once a week	883	63.8		
Smoking	Non-smoker	761	49.7		
	Smoker	775	50.3		
Parenting stress				27.40 ± 6.63	11–51
Daily working hours				8.04 ± 2.27	0–17.71
Psychological distress	Minimal	1187	77.2	5.26 ± 4.20	0–24
	Significant	345	22.8		
Involvement in childcare (quality)				14.46 ± 2.63	4–20
Involvement in childcare (quantity)				2.53 ± 1.62	0.14–14.86
Mother					
Psychological distress	Minimal	1069	69.4	5.79 ± 4.42	0–23
	Significant	467	30.3		
Child					
Age (months)				38.18 ± 1.51	35–42
Sex	Male	779	50.6		
	Female	762	49.4		
Temperament	Emotionality			14.35 ± 3.06	5–25
	Activity			19.14 ± 3.00	5–25
Family					
Household income	I (lowest)	341	23.2		
	II	369	27.0		
	III	392	27.4		
	IV (highest)	357	22.4		

No. of respondents is unweighted and percent (%) is weighted; M ± SD = Mean and standard deviation

The mean scores for paternal and maternal psychological distress were 5.26 ± 4.20 and 5.79 ± 4.42, respectively. The mean score of the quality of fathers’ involvement in childcare was 14.46 ± 2.63, and that of the quantity of fathers’ involvement was 2.53 ± 1.62.

### Differences in fathers’ involvement in childcare (quality/quantity) by covariates

Regarding the quality of fathers’ involvement in childcare, there were statistically significant differences in fathers’ education level (F = 10.02, *p* < .001), religion (t = − 2.476, *p* = .013), smoking (t = 4.379, *p* < .001), and family household income (*F* = 3.00, *p* = .030).

As for the quantity of fathers’ involvement in childcare, there were statistically significant differences in fathers’ education level (*F* = 5.95, *p* = .003), religion (t = − 3.545, *p* < .001), alcohol consumption (t = − 2.072, *p* = .038), and smoking (t = 4.559, *p* < .001) (Table 2).

**Table 2:** Differences in fathers’ involvement in childcare by characteristics (*N* = 1541)

Characteristics	Categories	Involvement in childcare (quality)		Involvement in childcare (quantity)	
		M ± SD	t or F(p)	M ± SD	t or F(p)
Father					
Education level	High school or less^a^	13.99 ± 2.77	10.02 (<.001) a < b,c	2.32 ± 1.62	5.95 (.003) a < c
	College^b^	14.48 ± 2.63		2.50 ± 1.63	
	University or higher^c^	14.69 ± 2.53		2.65 ± 1.61	
Religion	No	14.33 ± 2.62	−2.48 (.013)	2.41 ± 1.51	−3.55 (<.001)
	Yes	14.67 ± 2.64		2.72 ± 1.76	
Smoking	Non-smoker	14.76 ± 2.50	4.38 (<.001)	2.72 ± 1.67	4.56 (<.001)
	Smoker	14.17 ± 2.73		2.34 ± 1.55	
Alcohol consumption	More than once a week	14.30 ± 2.75	−1.39 (.165)	2.39 ± 1.55	−2.07 (.038)
	Less than once a week	14.51 ± 2.54		2.57 ± 1.59	
Child					
Sex	Male	14.40 ± 2.66	−0.10 (.344)	2.58 ± 1.74	1.15 (.250)
	Female	14.52 ± 2.61		2.48 ± 1.49	
Family					
Household income	I (lowest)^a^	14.20 ± 2.74	3.00 (.030) a < d	2.46 ± 1.79	1.56 (.209)
	II^b^	14.39 ± 2.57		2.48 ± 1.57	
	III^c^	14.47 ± 2.65		2.53 ± 1.45	
	IV (highest)^d^	14.78 ± 2.59		2.70 ± 1.71	

### Correlations between covariates, parental psychological distress, and fathers’ involvement in childcare (quality/quantity)

Table 3 shows the correlations between covariates, parental psychological distress, and fathers’ involvement in childcare (quality/quantity). Regarding the quality of fathers’ involvement in childcare, fathers’ age (*r* = −.062, *p* < .001), daily working hours (*r* = −.100, *p* < .001), parenting stress (*r* = −.358, *p* < .001), child’s age (*r* = .055, *p* = .030), temperament (emotionality) (*r* = −.093, *p* < .001), temperament (activity) (*r* = .062, *p* = .015), paternal psychological distress (*r* = −.236, *p* < .001), and maternal psychological distress (*r* = −.164, *p* < .001) significantly correlated with fathers’ involvement in childcare.

**Table 3:** Correlations between covariates, parental psychological distress, and fathers’ involvement in childcare (*N* = 1541)

Variable	Paternal psychological distress	Maternal psychological distress	Fathers’ involvement in childcare (quality)	Fathers’ involvement in childcare (quantity)
	r(*p*)	r(*p*)	r(*p*)	r(*p*)
Father’s age	.061 (.018)	.027 (.285)	−.062 (.016)	−.058 (.023)
Daily working hours	.086 (.001)	.104 (<.001)	−.100 (<.001)	−.297 (<.001)
Parenting stress	.432 (<.001)	.261 (<.001)	−.358 (<.001)	−.097 (<.001)
Child’s age	.001 (.968)	−.030 (.236)	.055 (.030)	−.022 (.399)
Temperament (emotionality)	.106 (<.001)	.274 (<.001)	−.093 (<.001)	−.047 (.066)
Temperament (activity)	−.078 (.002)	−.094 (<.001)	.062 (.015)	.018 (.479)
Paternal psychological distress	1	.385 (<.001)	−.236 (<.001)	−.073 (.004)
Maternal psychological distress		1	−.164 (<.001)	−.084 (.001)

As for the quantity of fathers’ involvement in childcare, fathers’ age (*r* = −.058, *p* = .023), daily working hours (*r* = −.297, *p* < .001), parenting stress (*r* = −.097, *p* < .001), paternal psychological distress (*r* = −.073, *p* = .004), and maternal psychological distress (*r* = −.084, *p* = .001) significantly correlated with fathers’ involvement in childcare.

### Moderating effect of maternal psychological distress

Table 4 shows the results of the moderating effect of maternal psychological distress on the influence of paternal psychological distress on fathers’ involvement in childcare (quality/quantity).

**Table 4:** Moderating effect of maternal psychological distress on the relationship between paternal psychological distress and fathers’ involvement in childcare (quality/quality) (*N* = 1541)

Variables	Dependent variable: Fathers’ involvement in childcare (quality)					
	Step 1		Step 2		Step 3	
	β	t(*p*)	β	t(*p*)	β	t(*p*)
Father’s age	−0.065	−2.57(.01)	−.059	−2.30(.020)	−.059	− 2.34(.020)
Education level	0.058	2.19(.029)	.056	2.11(.035)	.056	2.12(.035)
Religion	0.057	2.28(.023)	.057	2.26(.024)	.057	2.26(.024)
Smoking	−0.080	−3.14(.002)	−.075	−2.94(.003)	−.075	− 2.93(.003)
Daily working hours	−0.068	−2.67(.008)	−.061	− 2.38(.017)	−.061	− 2.37(.018)
Parenting stress	−0.339	−13.41(<.001)	−.302	−10.94(<.001)	−.301	− 10.92(<.001)
Child’s age	0.060	2.41(.016)	.06	2.43(.015)	.06	2.44(.015)
Temperament (Emotionality)	−0.042	−1.69(.092)	−.034	−1.31(.191)	−.034	− 1.31(.192)
Temperament (Activity)	0.034	1.35(.178)	.029	1.16(.246)	.029	1.15(.249)
Family household income	0.018	0.68(.499)	.014	0.54(.558)	.015	0.56(.573)
PPD			−.079	−2.74(.006)	−.085	−2.68(.007)
MPD			−.026	−0.92(.358)	−.03	−1.02(.309)
PPD*MPD					.014	0.44(.662)
R^2^	.170		.177		.177	
Adjusted R^2^	.164		.170		.169	
R^2^ change	.170		.001		<.001	
F(*p*)	28.193(<.001)		24.564(<.001)		22.676(<.001)	
Durbin-Watson test					2.006	
Variables	Dependent variable: Fathers’ involvement in childcare (quantity)					
	Step 1		Step 2		Step 3	
	β	t(*p*)	β	t(*p*)	β	t(*p*)
Father’s age	0.055	−2.12(.034)	−.053	−2.06(.04)	0.053	−2.06(.039)
Education level	0.028	1.04(.300)	.026	0.97(.335)	0.025	0.92(.357)
Religion	0.096	3.67(<.001)	.095	3.62(<.001)	0.095	3.62(<.001)
Smoking	0.044	−1.66(.096)	−.043	−1.62(.106)	0.044	−1.64(.101)
Alcohol consumption	0.062	−2.35(.019)	−.06	−2.28(.023)	0.058	−2.22(.026)
Daily working hours	0.309	11.73(<.001)	−.304	11.46(<.001)	0.305	11.49(<.001)
Parenting stress	−0.084	−3.25(.001)	−.069	−2.41(.016)	− 0.07	− 2.45(.015)
PPD			.006	0.19(.849)	0.024	0.71(.477)
MPD			−.07	−2.46(.014)	0.054	−1.77(.077)
PPD*MPD					0.043	−1.29(.196)
R^2^	.133		.137		.138	
Adjusted R^2^	.128		.131		.131	
R^2^ change	.133		.137		.001	
F(*p*)	28.584(<.001)		23.025(<.001)		20.901(<.001)	
Durbin-Watson test					1.844	

*PPD* Paternal Psychological Distress, *MPD* Maternal Psychological Distress

In the first step, father’s age, daily working hours, education level, religion, smoking, parenting stress; child’s age, emotionality, activity; and family household income were used as control variables. In the second step, the main effect of paternal psychological distress on fathers’ involvement in childcare (quality) was statistically significant (β = −.079, *p* = .006). Paternal psychological distress significantly increased fathers’ involvement in childcare (quality). In the last step, the interaction variables of paternal and maternal psychological distress did not significantly predict fathers’ involvement in childcare (quality) (β = .014, *p* = .662). Therefore, the moderation effect of maternal psychological distress on the relationship between paternal psychological distress and the quality of fathers’ involvement in childcare was not supported.

Regarding the quantity of fathers’ involvement in childcare, the main effect of paternal psychological distress on fathers’ involvement in childcare was statistically not significant (β = .006, *p* = .849). Therefore, the moderation effect of maternal psychological distress on the relationship between paternal psychological distress and the quantity of fathers’ involvement in childcare was not supported.

### Mediating effect of maternal psychological distress

A regression analysis was performed to verify the mediating effect of maternal psychological distress on the relationship between paternal psychological distress and fathers’ involvement in childcare (quality/quantity) (Table 5).

**Table 5:** Mediating effect of maternal psychological distress on the relationship between paternal psychological distress and fathers’ involvement in childcare (quality/quantity) (*N* = 1541)

Involvement in childcare (quality)								
Step	Independent variable	Dependent variable	B	SE	β	t(*p*)	R^2^	F(*p*)
Step 1	PPD	MPD	0.405	.025	0.385	16.344 (<.001)	.148	267.111 (<.001)
Step 2	PPD	Involvement in childcare (quality)	−0.148	.016	−0.236	−9.547 (<.001)	.056	91.150 (<.001)
Step 3	PPD	Involvement in childcare (quality)	−0.128	.017	−0.204	−7.626 (<.001)	.062	51.060 (<.001)
	MPD		−0.051	.016	−0.085	−3.182 (<.001)		
Sobel test: Z = 3.128, *p =* .002								
Involvement in childcare (quantity)								
Step	Independent variable	Dependent variable	B	SE	β	t(*p*)	R^2^	F(*p*)
Step 1	PPD	MPD	0.405	.025	0.385	16.344 (<.001)	.148	267.111 (<.001)
Step 2	PPD	Involvement in childcare (quantity)	−0.028	.010	−0.073	−2.884 (0.04)	.005	8.316 (.004)
Step 3	PPD	Involvement in childcare (quantity)	−0.019	.011	−0.048	−1.742 (.082)	.009	6.945 (.001)
	MPD		−0.024	.010	−0.065	−2.370 (.018)		
Sobel test: Z = 2.375, *p =* .018								

*PPD* Paternal Psychological Distress, *MPD* Maternal Psychological Distress

#### Quality of fathers’ involvement in childcare

In the first step, the regression analysis results indicated that the effect of paternal psychological distress on maternal psychological distress was significant (β = .385, *p* < .001), supporting paternal psychological distress as a reasonable explanation for 14.8% of variance in maternal psychological distress. In the second step, paternal psychological distress significantly affected the quality of fathers’ involvement in childcare (β = −.236, *p* < .001), supporting paternal psychological distress as a reasonable explanation for 5.6% of variance in the quality of fathers’ involvement in childcare. In the last step, both paternal psychological distress, as an independent variable, and maternal psychological distress, as the mediator, entered the regression model, with the quality of fathers’ involvement in childcare as a dependent variable. The results indicated that this relationship was significant (Z = 2.375, *p* = .018). Paternal psychological distress (β = −.204, *p* < .001) and maternal psychological distress (β = −.085, *p* < .001) were significant predictive variables of the quality of fathers’ involvement in childcare. The standardized regression coefficient of paternal psychological distress reduced from β = −.236 in the second step to −.204 in the third step, indicating a partial mediating effect of maternal psychological distress on the relationship between paternal psychological distress and the quality of fathers’ involvement in childcare. Finally, the Sobel test found that the mediating effect of maternal psychological distress on the relationship between paternal psychological distress and the quality of fathers’ involvement in childcare was significant (Z = 3.128, *p* = .002).

#### Quantity of fathers’ involvement in childcare

In the first step, the regression analysis results indicated that the effect of paternal psychological distress on maternal psychological distress was significant (β = .385, *p* < .001). In the second step, paternal psychological distress significantly affected the quantity of fathers’ involvement in childcare (β = −.073, *p* = .004). In the last step, both paternal psychological distress, as an independent variable, and maternal psychological distress, as the mediator, entered the regression model, with the quantity of fathers’ involvement in childcare as a dependent variable. The results indicated that only maternal psychological distress was a significant predictive variable of the quantity of fathers’ involvement in childcare (β = −.065, *p* = .018). In this model, the standardized regression coefficient of paternal psychological distress was not significant with respect to the quantity of fathers’ involvement in childcare (β = −.048, *p* = .082), indicating a complete mediating effect of maternal psychological distress on the relationship between paternal psychological distress and the quantity of fathers’ involvement in childcare. Finally, the Sobel test found that the mediating effect of maternal psychological distress on the relationship between paternal psychological distress and the quantity of fathers’ involvement in childcare was significant (Z = 2.375, *p* = .018).

## Discussion

Parenting requires the efforts of both parents, which is influenced by parents’ psychological state. This study investigated the relationship between parental psychological distress and involvement in childcare among fathers of preschool-aged children using the 2011 PSKC data. In contrast to previous studies [2], which primarily investigated parental psychological distress during the postpartum period only, this study investigated psychological distress in fathers of preschoolers, a period in which children are more vulnerable to the direct influence of parental psychological distress.

In the present study, 22.8% of fathers and 30.3% of mothers had significant psychological distress. This is higher than the results reported by a previous study for fathers (8.2%) and mothers (11.5%) with 3-year-old children [21]. However, this is similar to the results of previous research that reported that maternal psychological distress is higher than paternal psychological distress. Nevertheless, since psychological distress can get worse over time [22], identifying and treating psychological distress in both parents as early as possible is more cost-effective and can lead to better health outcomes [23]. Many countries provide screening and intervention for childbirth- and parenting-related psychological distress for women in early motherhood; however, early screening and intervention efforts for fathers—co-caregivers—who may be psychologically distressed is also important. Fathers’ psychosocial health not only affects their children’s and family’s current and future health and growth but also plays an important role in community health; as such, there is a need for efforts to detect paternal psychological distress early on and provide treatment.

In the present study, the quality and quantity of fathers’ involvement in childcare differed according to family characteristics. There was a statistically significant difference in the quality and quantity of fathers’ involvement in childcare depending on fathers’ education level, religion, smoking, and age. Further, the quantity of fathers’ involvement differed according to the frequency of fathers’ alcohol consumption, and the quality of fathers’ involvement varied as per household income.

As reported by Ishii-Kuntz [24], fathers’ age, education level, and income are associated with fathers’ involvement in childcare, indicating that socioeconomic disadvantage causes lower fathers’ involvement in childcare, ultimately influences children’s development. This can not only induce socioeconomic problems, even after the child grows up, but also adversely affect the society/community to which the child and family belong [25]. Thus, it is important to establish governmental support systems to encourage fathers’ involvement in childcare so as not to hurt children’s health and development.

Further, there was a positive correlation between fathers’ daily working hours and paternal and maternal psychological distress, and a negative correlation between daily working hours and the quality and quantity of fathers’ involvement in childcare. Bannai and Tamakoshi reported that long working hours adversely affect health outcomes, such as depression and psychological disorders [26]; furthermore, long working hours reduce the time available to spend with family, thereby hindering fathers’ ability to participate in childcare. Many countries endeavor to curtail weekly working hours, as such social endeavors are believed to help fathers maintain work–life balance, lower psychological distress, and secure time for their children and family.

Moreover, as fathers’ parenting stress increased, the quality and quantity of their involvement in childcare decreased. Parenting stress results in both short- and long-term negative consequences for both children and parents; in particular, for parents with depression or stress, parenting stress negatively affects their attitudes toward and their interaction with their children [27]. It is thus necessary to alleviate parenting stress by addressing the repetitive and routine stress that occurs in parenting.

Furthermore, there was a correlation between paternal and maternal psychological distress. In general, psychological disorders in married couples raising children are intimately associated [28, 29]. Because both fathers and mothers influence their children’s health and development, managing parents’ health, particularly their psychological distress, is important.

Higher psychological distress in fathers resulted in poorer quality and reduced quantity of their involvement in childcare. Psychologically distressed people are easily fatigued and tend to be indifferent to others [30]; as such, they may find it challenging to spend time with energetic and active preschoolers. Time spent on childcare during this stage of development, however, is crucial. Fathers can become more sensitive to their children’s personality and needs, learn parenting skills, and develop confidence as they interact with their children; therefore, the more the fathers spend time with their children, the more intimate their relationship becomes. Furthermore, fathers may have a positive or negative influence on their children depending on their attitude while they care for them; thus, how a father parents his child is also a critical component in the child’s development [2].

This study also analyzed the moderating and mediating effect of maternal psychological distress on the relationship between paternal psychological distress and fathers’ involvement in childcare. Maternal psychological distress was found to have a partial mediating effect between paternal psychological distress and the quality of fathers’ involvement in childcare, and a complete mediating effect between paternal psychological distress and the quantity of fathers’ involvement in childcare.

As there are no previous studies supporting the effect of maternal psychological distress on the relationship between paternal psychological distress and fathers’ involvement in childcare, an accurate comparison of the present results with previous research is difficult; however, maternal psychological distress has been reported to influence fathers’ involvement in childcare [31]. It is thus very important to reduce maternal psychological distress to guarantee the quality and quantity of fathers’ involvement in childcare. In addition, paternal psychological distress management is indispensable for increasing fathers’ involvement in childcare and for alleviating maternal psychological distress.

In this context, it is important to explore various means to qualitatively and quantitatively boost fathers’ involvement in childcare. To promote parental involvement in childcare, a family-centered approach for childcare should reflect the triadic interaction of father–mother–child. Healthcare providers in community childcare or childhood education centers need to perform an assessment of paternal as well as maternal psychological distress based on children’s developmental stages. To develop an intervention focused on parent–child relationships, primary healthcare professionals should consider father-centered sessions, including addressing stress management skills, to reduce work and parenting stress, in addition to improving parenting skills. Further, gender-specific learning preferences of fathers should be considered, such as by conducting evening or weekend educational programs.

This study, however, has several limitations. For example, it is a cross-sectional study (causality cannot be determined), and parental psychological distress was measured only for the past 4 weeks, although it has persistent and long-term effects on children. Therefore, further studies should consider adopting longitudinal designs to investigate the level of psychological distress among parents. Furthermore, the quality of fathers’ involvement in childcare was assessed using a scale that was completed by mothers; this should also be taken into consideration, because mothers’ evaluations are subjective. Future research should explore a means to objectively assess the quality of fathers’ involvement in childcare. Despite these limitations, this study provides meaningful contributions to the existing literature; it examines psychological distress and differences in it according to demographic characteristics among fathers of preschoolers, assesses the effect of maternal psychological distress on paternal psychological distress and fathers’ involvement in childcare, and determines the association between parental psychological distress and fathers’ involvement in childcare.

## Conclusion

In this study, paternal psychological distress was found to influence the quality and quantity of fathers’ involvement in childcare, and this was mediated by maternal psychological distress. Primary healthcare professionals, who play a key role in maternal and child health, should include screening for both maternal and paternal psychological distress when planning relevant programs. In addition to paying social attention to the psychosocial health of fathers, who are co-caregivers of their children, it is important for healthcare professionals to perform assessments and interventions for paternal psychological distress more actively in order to promote healthy parenting. This study enhances the understanding of fathers’ involvement in childcare through both qualitative and quantitative measurements, and the findings indirectly suggest the effects of parental mental health on childcare.

## Data Availability

All data are publicly available from http://panel.kicce.re.kr/eng/index.jsp.

## Abbreviation

PSKC: Panel Study on Korean Children
